# Clonal diversity of the glutamate dehydrogenase gene in *Giardia duodenalis *from Thai Isolates: evidence of genetic exchange or Mixed Infections?

**DOI:** 10.1186/1471-2180-11-206

**Published:** 2011-09-20

**Authors:** Suradej Siripattanapipong, Saovanee Leelayoova, Mathirut Mungthin, RC Andrew Thompson, Parima Boontanom, Wilai Saksirisampant, Peerapan Tan-ariya

**Affiliations:** 1 Department of Microbiology, Faculty of Science, Mahidol University, Rama VI Rd., Bangkok, 10400, Thailand; 2 Department of Parasitology, Phramongkutklao College of Medicine, Rajawithi Rd., Bangkok, 10400, Thailand; 3 WHO Collaborating Centre for the Molecular Epidemiology of Parasitic Infections, School of Veterinary and Biomedical Sciences, Murdoch University, South Street, Murdoch, Western Australia, 6150, Australia; 4 Department of Parasitology, Faculty of Medicine, Chulalongkorn University, Rama IV Rd., Bangkok, 10330, Thailand

**Keywords:** *Giardia duodenalis*, glutamate dehydrogenase, genetic diversity, genetic exchange

## Abstract

**Background:**

The glutamate dehydrogenase gene (*gdh*) is one of the most popular and useful genetic markers for the genotypic analysis of *Giardia duodenalis *(syn. *G. lamblia*, *G. intestinalis*), the protozoan that widely causes enteric disease in humans. To determine the distribution of genotypes of *G. duodenalis *in Thai populations and to investigate the extent of sequence variation at this locus, 42 fecal samples were collected from 3 regions of Thailand i.e., Central, Northern, and Eastern regions. All specimens were analyzed using PCR-based genotyping and recombinant subcloning methods.

**Results:**

The results showed that the prevalence of assemblages A and B among these populations was approximately equal, 20 (47.6%) and 22 (52.4%), respectively. Sequence analysis revealed that the nucleotide diversity of assemblage B was significantly greater than that in assemblage A. Among all assemblage B positive specimens, the allelic sequence divergence within isolates was detected. Nine isolates showed mixed alleles, ranged from three to nine distinct alleles per isolate. Statistical analysis demonstrated the occurrence of genetic recombination within subassemblages BIII and BIV was likely.

**Conclusion:**

This study supports increasing evidence that *G. duodenalis *has the potential for genetic exchange.

## Background

*Giardia duodenalis *(also known as *G. lamblia *and *G. intestinalis*) is a widely distributed diplomonad protozoon that causes enteric disease in humans and other vertebrates. This parasite has increasingly gained attention as a common cause of diarrheal disease in humans in both developed and developing countries. The average incidence of *G. duodenalis *is globally estimated at 2.8 × 10^8 ^cases each year [1]. In developing countries in Asia, Africa, and Latin America, approximately 200 million people are infected with this organism [2] with an average of 500,000 new cases per year [3]. Molecular studies have revealed that *G. duodenalis *is a morphologically uniform species complex [4-9]. Based on genetic data from the glutamate dehydrogenase (*gdh*) gene, a substantial level of genetic diversity in this species has been resolved into eight distinct lineages, assigned as assemblages A to H [2,10]. *G. duodenalis *recovered from humans falls only into assemblages A and B, which can be further classified into subgroups AI, AII, BIII, and BIV while the other assemblages (C to H) are animal-specific [2,10]. However, assemblages A and B have also been isolated from other animals, including livestock, cats, dogs, and rats.

*Giardia*, like other diplomonads, possesses two diploid nuclei (2 × 2N) in the trophozoite stage. Both nuclei, contain the same genetic information [11], are transcriptionally active [11,12] and replicate at approximately the same time [13]. On the other hand, in the cyst stage, the ploidy has changed to 16N (4 × 4N), which is the result of two rounds of nuclear division without cytokinesis [14,15]. Molecular data have revealed that certain nucleotides are different between the nuclei, with heterogeneity demonstrated between homologous chromosomes and allelic sequence heterozygosity (ASH). The level of ASH varies in different assemblages as assemblage B has been revealed to exhibit a higher level of overall ASH (0.5%) than that seen in assemblage A (< 0.01%) [16,17]. However, this low level of ASH is unusual for an asexually reproducing organism with a polyploid genome, like *Giardia*, indicating that some sort of genetic exchange may occur in and between trophozoites. One mechanism that can properly explain this finding is genetic recombination as a mean of maintaining a low level of ASH. Several studies have been conducted to provide more evidence of the existence of such a mechanism. Even though most studies supported the possibility of genetic recombination, the data were basically obtained from laboratory strains as well as small numbers of field isolates [18,19]. The aims of this study were to characterize nucleotide heterozygosity and provide more evidence of recombination within field isolates collected from different regions of Thailand using the *gdh *locus.

## Methods

### 1. Parasite isolates

A total of 42 fecal specimens of *G. duodenalis *were obtained from 3 regions of Thailand, as part of a public health survey. Each sample was coded with 2 or 3 letter codes to define the populations, 10 isolates with HT code were from the hill tribes, Northern Thailand, 19 isolates with Pre and TSH codes were from pre-school children and villagers in the Eastern part, and the 13 isolates with Or code were from orphans at a baby's home, Central Thailand.

*G. duodenalis *cysts were concentrated using a sodium nitrate flotation technique [20]. In brief, approximately 2 g of stools were suspended in 4 ml of 60% NaNO_3_, filtered through gauze and left for 20 minutes. One ml of the supernatant was collected from each sample then washed three times with phosphate buffered saline (PBS); the cysts in the sediment from the last wash were kept at -20°C until used.

### 2. Ethics statement

The ethical aspects of this study have been approved by the ethical committee of the Royal Thai Army Medical Department, Phramongkutklao College of Medicine, Thailand. Informed consent was written and was provided by all study participants and/or their legal guardians.

### 3. DNA preparation

DNA was extracted from concentrated stool samples using FTA Classic Card (Whatman Bioscience, USA). A total of 15 μl of concentrated stool was applied on a 6 mm-diameter FTA disk, and then was air-dried overnight. The one-fourth piece of FTA disk was washed twice with 200 μl of FTA purification reagent (Whatman Bioscience, USA) for 5 min and then washed twice with 200 μl of TE^-1 ^buffer (10 mM Tris-HCl, 0.1 mM EDTA [pH 8.0]) for 5 min and air-dried overnight. The washed paper was used directly as the DNA template in the PCR reactions. In addition, a QIAmp Stool Mini Kit (Qiagen, Germany) was used for DNA extraction for specimens that gave negative results with the FTA method.

### 4. DNA amplification

A nested PCR was performed to amplify a 456 bp fragment of the *gdh *gene by using primers and conditions previously described [21]. The primary PCR was carried out in a total volume of 25 μl reaction mixture containing 2 pieces of FTA disk or 1-2 μl of the extracted DNA as DNA template, 2.5 mM MgCl_2_, 250 mM of each deoxynucleoside triphosphate, 1 U of GoTaq DNA polymerase (Promega, USA) with 1× GoTaq PCR buffer, and 12.5 pmol of each primer, GDH1, GDH1a and GDH5s. Primary thermocycler conditions were as follows: (i) 7 min at 94°C; (ii) 35 cycles of 1 min at 94°C, 1 min at 55°C, and 1 min at 72°C; and (iii) 7 min at 72°C. The secondary PCR was carried out in a total volume of 25 μl reaction mixture that contained 2 to 5 μl of undiluted primary PCR product with the same concentrations as those of the primary PCR, except for 1.5 mM MgCl_2_, and GDHeF and GDHiR primers. The secondary thermocycler conditions were as follows: (i) 2 min at 94°C; (ii) 1 min at 56°C; (iii) 2 min at 72°C; (iv) 55 cycles of 30 sec at 94°C, 20 sec at 56°C, and 45 sec at 72°C; and (v) 7 min at 72°C. The amplified products were electrophoresed on a 1.25% agarose gel (Invitrogen, USA). DNA extracts of *G. duodenalis *from an axenic culture was used as positive control throughout the study.

### 5. DNA cloning and sequencing

The PCR products were purified using a Wizard^® ^SV Gel and PCR Clean-Up System (Promega, Madison, USA) according to the manufacturer's instruction and directly sequenced. Both strands of the entire fragments were sequenced with primers GDHeF and GDHiR, then manually assembled in BioEdit version 7.0.1. When the one singleton substitution was found, the sequencing was repeated with the PCR product from the independent PCR amplification. If a superimposed signal in chromatograms was detected, showing incorporation of the two bases resulting from co-amplification, cloning of this PCR product was performed to confirm the existence of the multiple templates. To clone, the purified PCR product was ligated into pGEM-T Easy vector (Promega, Madison, USA). Ligated product was introduced into JM109 competent cells by transformation. The recombinant plasmids were purified from 10 positive clones of each sample using the HiYield Plasmid mini kit (RBC Bioscience, Taiwan) and sequenced using universal primer SP6. DNA sequencing was conducted by 1^st ^Base Pte. Ltd., Singapore. The novel nucleotide substitutions obtained from clones corresponded to alleles if the substitution at that position occurred two or more times.

### 6. Sequence analysis

On all analyses, the priming sites were trimmed from both ends of all sequences which reduced the fragment size to 414 bp. All sequences were multiple aligned with the default option using CLUSTAL X, version 2.0.12 [22] and analyzed separately based on their assemblages, assemblage A and assemblage B. Each assemblage was both analyzed separately depending on the origins of the isolate and together. The partial sequences of the *gdh *gene of the *G. duodenalis *ATCC 50803 assemblage A isolate WB and *G. duodenalis *ATCC 50581 assemblage B isolate GS, acquired from *GiardiaDB: The Giardia Genomics Resource *http://giardiadb.org/giardiadb/, were used as reference sequences. The subassemblages were assigned through Bayesian inference constructed using MrBAYES Version 3.1.2 [23]. The reference sequences of assemblage AI (accession no. L40509), AII (accession no. L40510), BIII (accession no. AF069059), and BIV (accession no. L40508) were also implemented in the tree. The analysis of synonymous and non-synonymous amino acid substitutions was performed using MEGA version 4 [24]. The level of nucleotide divergence (K), including synonymous (Ks) and nonsynonymous (Ka) divergence rates, and number of allele were calculated using DnaSP version 5 [25]. This program was also used to quantify the level of genetic variation among *Giardia *isolates collected from different regions by the Wright's fixation index (*F*_ST_). This index is the correlation between alleles randomly chosen within the same population relative to alleles within the entire population. The effect of the amino acid substitutions was predicted based on sequence homology and the physical properties of amino acids using Sorting Intolerant From Tolerant (SIFT) program [26].

For distinguishing whether the fragments of DNA sequences were neutrally evolved or derived under selection processes, the Tajima's *D *was calculated using DnaSP version 5 [25]. Tajima's *D *statistic determines the difference between two nucleotide variation parameters, the average number of polymorphisms between all pairs of sequences (π) and the total number of polymorphic sites of all sequences in the dataset (θ). The greater value of π implies positive selection while the greater value of θ implies negative selection [27].

In order to test for recombination, *gdh *gene sequences of *G. duodenalis *available from GenBank on March 2010 were additionally included in the analysis. Because the region and the length of the *gdh *sequences deposited in GenBank varied depending on the primers used by individual research studies, the 75 sequences originated from 14 countries were selected with the minimum coverage at 75% to the fragment size used for analysis in this study (Table 1). The phylogenetic network tree was used to visualize the extent of networked evolution among the sequences which preliminarily indicate possible locations of recombination events [28]. Principally, the phylogenetic tree and phylogenetic network tree are each constructed on a different basis. The phylogenetic tree is constructed under the assumption that once two lineages are created, they will subsequently not interact with each other again, whereas the phylogenetic network assumes the evolutionary process in a more relaxed manner and constructs the tree under the assumption that the interaction between these two lineages might have occurred again later on. To present the data according to the aims of this study, this method is more appropriate than a conventional bifurcating phylogenetic tree. The analysis was undertaken with the SplitsTree program version 4 [29], through the Neighbor-Net method [30]. This method draws networks between sequences if there are potentially multiple evolutionary pathways linking them. The analysis was performed using sequences of all isolates presented in this study together with the sequences selected from GenBank. For the isolates that carried the heterozygous polymorphic sites identified by cloning, the standard one-letter code for combining nucleotides defined by the International Union of Pure and Applied Chemistry nomenclature (IUPAC) was used.

**Table 1:** Characteristics and sources of the isolates from GenBank

**No**.	**Accession No**.	Isolates	Assemblage	Geographical origin	% coverage
1	EU594667.1	Cub-G81	BIII	Cuba	100
2	EU594666.1	Cub-G12	BIV	Cuba	100
3	EU594665.1	Cub-G89	BIII	Cuba	100
4	EU594664.1	Cub-G33	BIII	Cuba	100
5	EU594663.1	Cub-G91	B	Cuba	100
6	EF507682.1	H43	BIV	Sao Paulo	100
7	EF507672.1	H30	BIV	Sao Paulo	100
8	EF507671.1	H29	BIV	Sao Paulo	100
9	EF507668.1	H25	BIV	Sao Paulo	100
10	EF507665.1	H22	BIV	Sao Paulo	100
11	EF507664.1	H21	BIV	Sao Paulo	100
12	EF507646.1	H1	BIV	Sao Paulo	100
13	DQ840541.1	gi-hum1	BIV	Poland	100
14	DQ090541.1	gd-ber10	BIII	Norway	100
15	DQ090540.1	gd-ber9	BIII	Norway	100
16	DQ090539.1	gd-ber8	BIV	Norway	100
17	DQ090538.1	gd-ber7	BIII	Norway	100
18	DQ090537.1	gd-ber6	BIII	Norway	100
19	DQ090536.1	gd-ber5	BIII	Norway	100
20	DQ090535.1	gd-ber4	BIII	Norway	100
21	DQ090534.1	gd-ber3	BIV	Norway	100
22	DQ090533.1	gd-ber2	BIII	Norway	100
23	DQ090532.1	gd-ber1	BIII	Norway	100
24	DQ923589.1	gd-ber20	BIII	Norway	100
25	DQ923588.1	gd-ber19	BIII	Norway	100
26	DQ923586.1	gd-ber17	BIV	Norway	100
27	DQ923585.1	gd-ber16	BIV	Norway	100
28	DQ923584.1	gd-ber15	BIII	Norway	100
29	DQ923583.1	gd-ber14	BIII	Norway	100
30	DQ923582.1	gd-ber13	BIV	Norway	100
31	DQ923581.1	gd-ber12	BIV	Norway	100
32	DQ923580.1	gd-ber11	BIII	Norway	100
33	AY826197.1	NLH35	BIV	Dutch	100
34	AY826193.1	NLH25	BIV	Dutch	100
35	AY826192.1	NLH28	BIV	Dutch	100
36	AY826191.1	NLH13	BIV	Dutch	100
37	AY178756.1	FCQ-21	BIII	Mexico	100
38	AF069059.1	BAH-12	BIII	Australia	100
39	L40508.1	Ad-7	BIV	Australia	100
40	AY178739.1	Ad-45	BIV	Australia	100
41	AY178738.1	Ad-28	BIV	Australia	100
42	AY178755.1	Ad-85	BIV	Australia	100
43	AY178754.1	Ad-82	BIV	Australia	100
44	AB295654.1	PalH8-3	BIII	Palestine	94.4
45	AB295653.1	PalH8-2	BIV	Palestine	94.4
46	AB295652.1	PalH8-1	BIII	Palestine	94.4
47	AB295651.1	PalH4-3	BIV	Palestine	94.4
48	AB295650.1	PalH4-2	BIV	Palestine	94.4
49	AB295649.1	PalH4-1	BIII	Palestine	94.4
50	AB479246.1	NplH9	BIII	Nepal	76.8
51	AB479245.1	NplH8	BIII	Nepal	76.8
52	AB479244.1	NplH6	BIV	Nepal	76.8
53	AB479243.1	NplH5	BIII	Nepal	76.8
54	AB479242.1	NplH4	BIV	Nepal	76.8
55	AB479241.1	NplH1	BIII	Nepal	76.8
56	AB479121.1	Nepal	BIII	Nepal	76.8
57	AB479240.1	JpnH5	BIII	India	76.8
58	AB479239.1	JpnH1	BIII	Burkina Faso	76.8
59	AB479238.1	IdnH40	BIII	Indonesia	76.8
60	AB479237.1	IdnH39	BIII	Indonesia	76.8
61	AB479248.1	IdnH5	BIV	Indonesia	76.8
62	AB479247.1	IdnH3	BIV	Indonesia	76.8
63	AB479236.1	IdnH37	BIII	Indonesia	76.8
64	AB479235.1	IdnH28	B	Indonesia	76.8
65	AB479234.1	IdnH25	BIV	Indonesia	76.8
66	AB479233.1	IdnH24	BIV	Indonesia	76.8
67	AB479232.1	IdnH21	BIII	Indonesia	76.8
68	AB479231.1	IdnH18	BIV	Indonesia	76.8
69	AB479230.1	IdnH17-2	BIV	Indonesia	76.8
70	AB479228.1	IdnH14	BIV	Indonesia	76.8
71	AB195224.1	GH-135	BIII	Japan	100
72	AB182126.1	GH-156	BIV	Japan	100
73	AB188825.1	GH-158	BIV	Japan	100
74	AB434535.1	TIG12	BIII	Iran	100
75	AB434534.1	TIG7	BIII	Iran	91.1

To provide the evidence on recombination that could occur, the alignments were examined using two tests: the four-gamete test from the DnaSP version 5 [25] and the Φ statistic test from the PhiPack program [31]. The four-gamete test is the method based on the principle of compatibility that has the number of recombination events as the quantity of interest. This method functions under the infinite-alleles model in which the mutation rate for any site is infinitesimal and only the mutation would lead to the different alleles. As such, when considering any two sites, there are at most four gametic types in the population. Since the back mutation and recurrent mutation is negligible in this model, the presence of all four gametic types will be due to the occurrence of recombination event between the two sites [32]. In PhiPack, the Φ (or pairwise homoplasy index, PHI) statistic, the method based on refined incompatibility, is used to detect the recombination. This test relies on the assumption that the level of genealogical correlation between neighboring sites is negatively correlated with the rate of recombination [31]. If the recombination rate is zero, all sites have the same history and the order of the sites does not reflect the genealogical correlation. On the other hand, if the recombination rate is finite, the order of the sites becomes important as distant sites give a tendency to have less genealogical correlation than adjacent sites. The significance of the analysis is obtained using a permutation test. In this study, the parameters were set to examine the significance of the test using 1000 PHI permutation and window size at 100.

### 7. Sequence data

Sequences from isolates generated in this study were deposited in the GenBank database under accession no. HM747962-HM748047.

## Results

### Diversity of the isolates

Determination of the 414 bp region of the *gdh *gene obtained from direct sequencing revealed that, among the 42 isolates, clear electrochromatograms without any superimposed signals were observed in 33 (78.6%) isolates. Of the remaining nine (21.4%) isolates, multiple signals were observed in certain positions along the sequences. Subcloning and sequencing of these isolates making up the whole dataset contained 54 distinct alleles from a total 86 isolates/clones. The multiple alleles held by each isolate ranged from three to nine alleles; nine different alleles in isolate Pre2403, eight alleles in isolate Or172 and Pre1402, seven alleles in isolate HT187, five alleles in isolate HT57 and HT105, four alleles in isolate HT193 and Pre2103, and three alleles in Or176 (Table 2 and 3).

**Table 2:** The variable sites alignment of *gdh *gene fragment of *G.duodenalis *in 20 isolates of assemblage A.

	**2266**
**Isolates**	**3402**
	**7631**
ATCC50803	CCTC
HT124	..CT
HT137	..CT
HT144	..CT
Or006	..CT
Or019	..CT
Or140	..CT
Or215	..CT
Or262	..CT
Or287	..CT
Or87	..CT
Or88	..CT
Or94	..CT
Or98	..CT
Pre1209	..CT
Pre2208	..CT
Pre3111	TTCT
TSH1123	..CT
TSH2014	..CT
TSH292	..CT
TSH408	..CT

Amino acid	VNSA ....

Dots are identical sites. Numbers indicate nucleotide positions from start codon.

**Table 3:** The variable sites alignment of *gdh *gene fragment of *G.duodenalis *in 22 isolates of assemblage B.

	**2333333334**	**4445555555**	**55556666**
**Isolates/clones**	**7034568992**	**3450014566**	**67790122**
	**4990707169**	**6761790814**	**70676247**
ATCC50581	CTGACGCGTC	TCGCCCTGTG	CCGCCAAG
HT105	..........	........C.	........
HT123C1	..........	........C.	...T....
HT123C10	....T.....	......C.C.	.....G..
HT123C2	....T.....	........C.	...T....
HT123C4	....T.....	........C.	.T.T....
HT123C7	..........	........C.	.T.T....
HT140	......T...	........C.	........
HT142	..........	..........	........
HT187C1	..........	.T.....AC.	........
HT187C2	..........	........C.	........
HT187C3	....T.....	.T.....AC.	.....G..
HT187C4	....T.....	..........	....T..A
HT187C5	....T.....	....T.....	....T..A
HT187C6	....T.....	.T.....AC.	....T..A
HT187C8	....T.....	........C.	........
HT193C1	....T.....	..........	..A.....
HT193C2	..........	..........	..A.....
HT193C8	....T.....	....T.....	....T..A
HT193C9	..........	......C.C.	.....G..
HT57C1	..........	..........	........
HT57C2	..........	..A.....C.	.T......
HT57C3	..........	........C.	.T......
HT57C5	...G......	G.........	........
HT57C8	..........	G.........	........
Or172C1	.C..T....T	.T...TC.C.	.....G..
Or172C2	.........T	........C.	....T...
Or172C3	G..G......	.T...TC.C.	.....G..
Or172C4	....T.....	........C.	........
Or172C5	........C.	........C.	.....G..
Or172C6	.C..T....T	.T......C.	........
Or172C7	..........	.T...TC.C.	.....G..
Or172C8	.........T	.T...TC.C.	.....G..
Or176C1	..........	........C.	........
Or176C2	..........	........C.	....T...
Or176C9	........C.	........C.	....T...
Or284	....T.....	..........	....T...
Pre016	..........	........C.	........
Pre1117	.C..T....T	.....TC.CA	.....G..
Pre1402C1	..........	..........	....T..A
Pre1402C2	....T.....	........C.	....T..A
Pre1402C4	.......A..	....T...C.	.......A
Pre1402C5	..........	........C.	........
Pre1402C6	.......A..	........C.	........
Pre1402C7	....T..A..	.......AC.	........
Pre1402C8	....T.....	........C.	....T...
Pre1402C9	....T..A..	....T...C.	....T...
Pre2018	..........	........C.	........
Pre2103C1	..........	..A.....C.	........
Pre2103C2	..........	..A.....C.	T.......
Pre2103C3	..........	........C.	........
Pre2103C5	..........	........C.	T.......
Pre2320	....T.....	....T.....	....T..A
Pre2403C1	..........	.....TC.C.	...T..G.
Pre2403C10	.........T	.....T..C.	.T..T...
Pre2403C2	G.........	...T....C.	........
Pre2403C3	.C...A....	........C.	....T...
Pre2403C4	..A..A....	........C.	.T..T...
Pre2403C5	.........T	........C.	.T..T...
Pre2403C6	..........	...T....C.	.T......
Pre2403C7	..A..A...T	.....TC.C.	...T..G.
Pre2403C8	.........T	.....TC.C.	...T..G.
Pre3207	..........	........C.	......G.
TSH090	..........	..A.....C.	........
TSH1119	..........	..A.....C.	........
TSH1210	..........	........C.	...T....
TSH1250	..........	........C.	........

Amino Acid	LLKNLPDDPF	STQGGIYEFT	GVTGFRTG
..........	V..D...N..	A.........	........

Dots are identical sites. Numbers indicate nucleotide positions from start codon.

### Assemblage assignment

**Figure 1:**
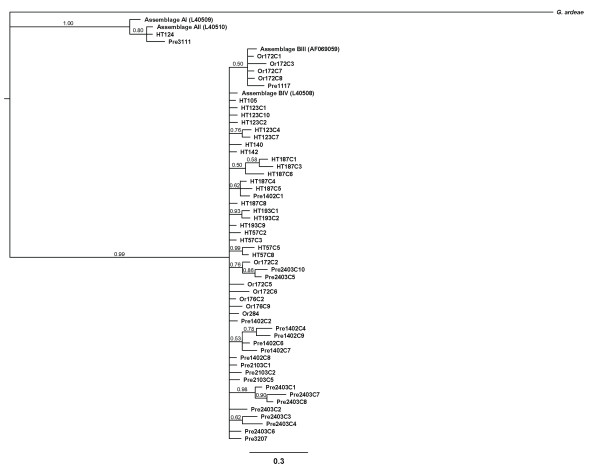
**Bayesian analyses of the *gdh *gene were performed using the HKY85+Γ+I, selected by jModelTest version 0.1 **[42]**, as a model of sequence evolution**. Starting trees were random, four simultaneous Markov chains were run for 1,000,000 generations, and trees were sampled every 100 generations. Bayesian posterior probabilities were calculated using a Markov chain Monte Carlo sampling approach implemented in MrBAYES program. The sequence HT124 is 100% identical to HT137, HT144, Or006, Or019, Or87, Or88, Or94, Or98, Or140, Or215, Or262, Or287, Pre1209, Pre2208, TSH292, TSH408, TSH1123, and TSH2014. The sequence HT105 is 100% identical to HT187C2, Or176C1, Pre016, Pre1402C5, Pre2018, Pre2103C3, and TSH1250. The sequence HT123C1 is 100% identical to TSH1210. The sequence HT142 is 100% identical to HT57C1. The sequence HT187C5 is 100% identical to HT193C8 and Pre2320. The sequence HT187C8 is 100% identical to Or172C4. The sequence Pre2103C1 is 100% identical to TSH090 and TSH1119. Posterior probabilities < 0.50 are omitted.

As shown in Figure 1, the Bayesian inferred tree based on the distance method showed that the *gdh *sequences of all 86 isolates/clones were clustered into two major clades with the respective reference sequences, assemblages A and B. Within the assemblage A clade, using HT124 and HT105 as representatives, 20 isolates were clustered with the assemblage AII reference sequence while none belonged to assemblage AI. The remaining 66 sequences/clones from 22 isolates were placed in the assemblage B clade which divided into two sister clades. Five sequences/clones from two isolates were grouped in the subclade belonging to the subassemblage BIII and the other 61 sequences/clones from 21 isolates were clustered within subassemblage BIV subclade. These results showed that prevalence of the isolates carrying assemblages A and B was approximately equal, 47.6% and 52.4%, respectively, and the prevalence of subassemblage BIV was predominant over subassemblage BIII. Moreover, the phylogenetic analyses also showed that four of eight distinct clones obtained from isolate Or172 were assigned to subassemblage BIII (clones C1, C3, C7, and C8) whereas clones C2, C4, C5, and C6 shared a closer relationship to subassemblage BIV.

### Sequence variation and allelic divergence

Analysis of 20 assemblage A isolates revealed that few variations occurred within this assemblage. Only two different alleles were observed with four synonymous substitutions when compared with the reference sequence. No sequence variation was found within this group except for the single different allele from the isolate Pre3111 that contained two different sites. The overall intra-assemblage divergence of this assemblage (K) was only 0.96% and the divergence at synonymous positions (Ks) was 0.0019. In assemblage B, the 66 sequences/clones showed that they were 52 different alleles with 4 nonsynonymous and 24 synonymous amino acid substitutions when compared with their reference sequence. The intra-assemblage variation of this assemblage was 6.76% with the divergence of synonymous (Ks) and nonsynonymous positions (Ka) at 0.039 and 0.001, respectively (Table 4). Due to the nucleotide substitutions, four nonsynonymous changes were detected. Thus, nucleotide changes at position 274, Leucine (L) was changed to Valine (V). At position 340, Asparagine (N) was changed to Aspartic acid (D) while at position 391, Aspartic acid (D) was changed to Asparagine (N) and at position 436, Serine (S) was changed to Alanine (A) (Table 5). The SIFT software was used to predict the effect of these changes with 41 homologous sequences fetched from the UniProt-SwissProt 56.6 database. Using SIFT, it predicts the possibility of the effect caused by the substitution change by using the scoring method. The score is the normalized probability that the amino acid change is tolerated. The reliability of this score is supported by the value, which measures the diversity of the sequences in the alignment. Generally, the substitution site of the score less than 0.05 is predicted as a deleterious site with the support of median conservation values between 2.75 and 3.25 considered as a reliable prediction. Our results showed that all substitution changes were tolerated to the alteration of the protein function with all prediction scores > 0.05 and supported by the median conservation value of 3.08 (Table 5).

**Table 4:** The genetic divergence of assemblages A and B

Assemblage	Nucleotide divergence (%)	Ks	Ka
A	0.96	0.0019	-^a^
B	6.76	0.039	0.001

Ks; divergence at synonymous positions, Ka; divergence at nonsynonymous positions;^a^no nonsynonymous change

**Table 5:** Score and median conservation values from the prediction of the effect of amino acid substitutions

Positions	Substitution Changes	Score	Median conservation
274	Leu to Val	0.34	3.08
340	Asn to Asp	0.11	3.08
391	Asp to Asn	0.1	3.08
436	Ser to Ala	1.0	3.08

Since the low genetic variation level of assemblage A does not reach the usual value observed in sexual populations, almost identical nucleotide sequences do not warrant further analysis. Thus, the sequence data of assemblage A were not included in the downstream analysis.

### Estimate of geographic differentiation

Phylogenetic analysis has shown that both assemblage A and B isolates have been dispersed throughout all studied geographical locations and appeared to be weakly supported for geographical sub-structuring. To determine if the traits from this inference were correct, the level of genetic distinction between each geographic population was estimated. The Wright's test measures the level of genetic distinction between populations, representing with fixation index (*F*_ST_) value from 0 to 1. A value of zero indicates no divergence and implies that two populations are freely spread whereas the positive deviation from zero indicates the extent of genetic differences. A value of one would imply that two populations are completely separate. The estimated values showed little difference between each pair of the three regions and no significant differences were exhibited (Table 6). The overall results from these analyses implied that the same population of parasite isolates spread throughout all these studied areas.

**Table 6:** The level of genetic distinction between each pair of different populations (northern, eastern, and central)

Assemblage/Populations	Level of genetic distinction	
	
	*F*_ST_	*P*-value
B/northern vs B/central	0.132	0.44
B/northern vs B/eastern	0.044	0.36
B/central vs B/eastern	0.103	0.31

### Test for neutrality and recombination

The values of Tajima's *D *statistical estimation are shown in Table 7. Across all populations and in each population, the test gave a tendency for negative values that is indicative of the occurrence of selection pressure. However, these results were not statistically significant (Table 7).

**Table 7:** Test for neutrality for all populations, northern, central, eastern, and plus all sequences from GenBank

Assemblage/Populations	Tajima's *D*
B/All	-0.83636
B/northern	-0.46236
B/central	-0.65253
B/eastern	-0.79615
B/All+GenBank	-^a^

^a^Not analyzed

**Figure 2:**
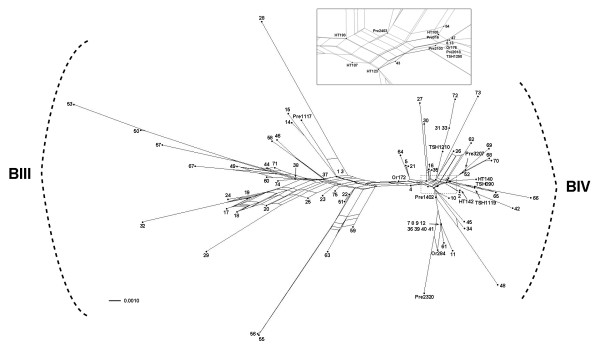
**Phylogenetic network was built by Neighbor-Net using *gdh *sequence fragments from this study and from those of GenBank**. The numbers labeled in the network are from Table 1. The magnified image in the closed box shows details of the area covered by dotted box.

For the test of recombination, the phylogenetic network reconstructed from the *gdh *gene fragment obtained in this study and GenBank partially gave a treelike structure, except the area at the center of the tree. The network was separated into two large branches, according to subassemblages BIII and BIV, with long and short branches extending from both of them (Figure 2). The conflicting signals were explicitly observed in both branches, which implied the alternative phylogenetic histories existed separately existed in both subassemblages. Of 75 sequences from 14 countries, they seemingly dispersed throughout both branches with no specific geographical significances observed. Additionally, the four-gamete test detected recombination events within the sequence data of this study in both subassemblages BIII and BIV, suggesting intra-assemblage recombination among them. In addition, the same results still persisted when the sequence data from GenBank were additionally included in the test. The significance of recombination identified by the four-gamete test was further emphasized with the additional implementation of the Φ test. The results from this test were almost consistent to the former test and showed statistical significances within all dataset, except for the data of subassemblage BIV from this study alone (Table 8).

**Table 8:** Test for recombination for subassemblages BIII and BIV using dataset of this study and dataset of this study plus dataset from GenBank

Assemblage/Dataset	Four-gamete^a^	Φ
BIII/this study	Yes	Yes*
BIV/this study	Yes	No
BIII/this study+GenBank	Yes	Yes*
BIV/this study+GenBank	Yes	Yes*

^a^The test does not assign significance

*P < 0.01

## Discussion

This study focused on genetic diversity of *G. duodenalis *at the *gdh *gene using isolates collected from three different regions of Thailand. Cloning and sequencing approaches were used to elucidate heterologous alleles existed within the samples. Many studies have often detected overlapping nucleotide peaks which represented as mixed template at several genetic markers from different geographical locations [33]. The result of mixed templates gives rise to a question whether this phenomenon is actually the result of mixed infection or the occurrence of ASH. Until now, there is still no direct evidence to prove which one plays a major role in the occurrence of ambiguous nucleotides. Thus, to provide conclusive evidence, further studies are required to explain the existence of ASH using cloned isolates of *G. duodenalis *which has never been shown by any studies. Although our study used the isolates from the patients without being cloned, to support the existence of ASH, indirect evidence of genetic exchange by recombination was obtained using bioinformatics studies.

The results obtained from the present study revealed that *G. duodenalis *isolates containing multiple alleles naturally presented in every area surveyed in Thailand, as shown by sequencing results of the subclones from isolates having overlapping chromatogram signals. These heterogenous sequencing results were observed only within assemblage B and throughout subtypes BIII and BIV whereas all assemblage A was homogeneous. The co-amplification of the cross-contaminated isolate was unlikely to occur because the isolates from each region were collected and processed at different times. Additionally, every isolate that revealed mixed templates was repeatedly tested under independent PCR and sequencing reactions. However, this finding seems to be common, as the occurrence of heterogeneous positions in the sequences of the *gdh *gene of assemblage A is markedly low [34]. The presence of heterogenous nucleotides obtained from direct sequencing is usually considered to be the results of simultaneous infection with more than one *Giardia *assemblage. However, using the subcloning technique, the abundance of nine different *gdh *alleles observed in some isolates, lead us to presume that it could not be only the outcome of mixed infection. Hence, the existence of the ASH in these isolates should also be taken into consideration.

Alignment analysis of the polymorphic sites within assemblage B revealed that almost all nucleotide substitutions observed were synonymous changes, except for four positions. The Tajima's *D *test on the *gdh *gene showed contrasting results to those obtained with the β-giardin gene of other studies. The β-giardin gene was likely to be under the effects of ongoing purifying selection [35] while the *gdh *gene was under neutral selection. This suggested that molecular adaptation of these two genes might be influenced by different pressures. Furthermore, the computational prediction estimated that these changes did not influence the protein function. It indicated that variations appeared in the amino acid level are neutral or advantageous but not deleterious. The prediction is based on the non-structure method that considers the information from the amino acid sequence of interest, such as the position and type of amino acid changes, and compares their properties with the homolog protein family in the database [26]. This method seems to be the most reliable option to predict the effect of the nonsynonymous substitution in this gene since most of the *gdh *gene studies are based on partial sequences. This may be due to the limitation of primer design to amplify the whole gene as this gene contains a number of variations and high percentage of GC content [36].

The estimation of the fixation index between three different sampling areas in Thailand did not support geographical sub-structuring within the *G. duodenalis *isolates. At present, the variations found in this study could not explain the geographical distribution of infected individuals. The only observation about the geographical aspect shown in this study is that the *G. duodenalis *populations were widely distributes throughout all three regions. The lack of geographical sub-structuring shown in this study was not unexpected since small fragments of only one gene were used to analyze the geographical distribution of this protozoan. Nevertheless, to the best of our knowledge, there is still no genotyping system that can efficiently indicate geographical sub-structuring of this organism, even using multilocus genes as genotypic markers [37]. Whilst, the application of the high-resolution genotyping system is still necessary to address this question since it will be useful to distinguish different transmission routes and sources of infection.

Since the first finding of the genes known to function during meiosis and later confirmed by cloning and sequencing of PCR products [19,38], the question about the potential capability of sexual reproduction in *Giardia *has been proposed. Subsequently, a number of studies have been conducted to provide evidence in support of genetic exchange among *G. duodenalis *isolates [18,19,39]. The present research attempted to extend the study of this issue to the next step by testing the potential of recombination events with the genetic data obtained from field isolates. In this study, we used the recombination analysis to show that the ASH could be a consequence of genetic exchange.

When the reticulate events, such as hybridization, gene transfer, and genetic recombination, are suspected to be involved, the phylogenetic network is one of the method that play a role in the accommodation of the non-treelike evolution. By using an agglomerative process implemented in the algorithm of Neighbor-Net, it can represent the conflicting signal or alternative phylogenetic histories, which are not adequately modeled by the bifurcating phylogenetic tree, in the format of a split graph. The presentation of the reticulations in the network indicated the possibility of interaction between two hypothetical ancestors to become extant taxa. In this study, the network tree clearly showed that the recombination might not be a phenomenon limited to laboratory strains and the interactions between taxa separately occurred within their own lineages of assemblages BIII and BIV.

Besides the evidence from the phylogenetic network tree, more intensive analyses were applied to further investigate the possibility of recombination from the dataset of this study. Two tests were selected based on their different assumptions for detecting the recombination to validate the evidence obtained from network tree. Four-gamete test is different from other general recombination testing methods that it is the population-specific method, generating to detect recombination between closely related genotypes. However, not all recombination events are revealed by this test due to its limitation that not support the occurrence of the recurrent or convergent mutations.

To confirm the results from the four-gamete test, a robust statistical test for recombination, Φ test, was applied. This recently developed approach is designed to operates under more relax model and has been proved through empirical data analysis that it can effectively discriminate between the presence and absence of recombination in both closely and distantly related samples [31].

The positivity of the four-gamete test and the statistical significance obtained from the Φ test strongly indicated the existence of the recombination in both subassemblages BIII and BIV. However, the recombination events were not significant when analyzing only sequence data of subassemblage BIV. This might be due to a small number of sequence data used for analysis (only 5 sequences tested). Low levels of variation among sequences limited the detection of recombination using this test [40]. Generally, there are four major goals in the study of recombination that are i) detecting evidence of recombination in a dataset, ii) identifying the mosaic sequences, iii) delineating their breakpoints, and iv) quantifying recombination [41]. Clearly, the majority of the *Giardia *studies, including this study, are in the early stage for recombination analysis that all evidences are indirectly detected from the mathematical and statistical models. Usually, if significant evidence for recombination can be detected, the localization of the recombination breakpoint is the next goal for the analysis. If the mosaic pattern of the sequence can be demonstrated, this will support the existence of genetic recombination in this organism.

## Conclusions

We demonstrated that some field isolates of *G. duodenalis *from Thailand contained heterogeneity and sequence variations, especially those of assemblage B. The alternative evolution signals visualized by the phylogenetic network tree with the association of the results from two powerful recombination tests strongly supported evidence of genetic exchange by recombination in this organism.

## Authors' contributions

SS participated in the study design, carried out most of experiments, analyzed and interpreted the data, and co-wrote the manuscript. SL, MM, and AT participated in the study design, supervised the experiments, and co-wrote the manuscript. WS participated in specimen collection. PB participated in DNA extraction. PT conceived the project, supervised the experiments and co-wrote the manuscript. All authors read and approved the final manuscript.
